# Spectral imaging toolbox: segmentation, hyperstack reconstruction, and batch processing of spectral images for the determination of cell and model membrane lipid order

**DOI:** 10.1186/s12859-017-1656-2

**Published:** 2017-05-12

**Authors:** Miles Aron, Richard Browning, Dario Carugo, Erdinc Sezgin, Jorge Bernardino de la Serna, Christian Eggeling, Eleanor Stride

**Affiliations:** 1 Department of Engineering Science, Institute of Biomedical Engineering, University of Oxford, Oxford, OX3 7DQ UK; 2 Faculty of Engineering and The Environment, University of Southampton, Southampton, SO17 1BJ UK; 3 MRC Human Immunology Unit, Weatherall Institute of Molecular Medicine, University of Oxford, Headley Way, Oxford, OX3 9DS UK; 4 Research Complex at Harwell, Central Laser Facility, Rutherford Appleton Laboratory, Science and Technology Facilities Council, Harwell-Oxford, OX11 0FA UK

**Keywords:** Spectral imaging, Lipid order, Lipid packing, Membrane viscosity, Membrane segmentation, Laurdan

## Abstract

**Background:**

Spectral imaging with polarity-sensitive fluorescent probes enables the quantification of cell and model membrane physical properties, including local hydration, fluidity, and lateral lipid packing, usually characterized by the generalized polarization (GP) parameter. With the development of commercial microscopes equipped with spectral detectors, spectral imaging has become a convenient and powerful technique for measuring GP and other membrane properties. The existing tools for spectral image processing, however, are insufficient for processing the large data sets afforded by this technological advancement, and are unsuitable for processing images acquired with rapidly internalized fluorescent probes.

**Results:**

Here we present a MATLAB spectral imaging toolbox with the aim of overcoming these limitations. In addition to common operations, such as the calculation of distributions of GP values, generation of pseudo-colored GP maps, and spectral analysis, a key highlight of this tool is reliable membrane segmentation for probes that are rapidly internalized. Furthermore, handling for hyperstacks, 3D reconstruction and batch processing facilitates analysis of data sets generated by time series, z-stack, and area scan microscope operations. Finally, the object size distribution is determined, which can provide insight into the mechanisms underlying changes in membrane properties and is desirable for e.g. studies involving model membranes and surfactant coated particles. Analysis is demonstrated for cell membranes, cell-derived vesicles, model membranes, and microbubbles with environmentally-sensitive probes Laurdan, carboxyl-modified Laurdan (C-Laurdan), Di-4-ANEPPDHQ, and Di-4-AN(F)EPPTEA (FE), for quantification of the local lateral density of lipids or lipid packing.

**Conclusions:**

The Spectral Imaging Toolbox is a powerful tool for the segmentation and processing of large spectral imaging datasets with a reliable method for membrane segmentation and no ability in programming required. The Spectral Imaging Toolbox can be downloaded from https://uk.mathworks.com/matlabcentral/fileexchange/62617-spectral-imaging-toolbox.

## Background

An increasing body of evidence suggests that the dynamic reorganization of lipids in cellular membranes can compartmentalize membrane proteins, influencing a cell’s response to extracellular stimuli and its membrane permeability [1, 2]. It follows that drug-carrying agents, such as liposomes or gas microbubbles, with optimized lipid compositions can exploit these processes for enhanced drug-delivery via membrane fusion or membrane permeabilization [3–5]. To facilitate the characterization of such drug-delivery devices and to deepen our understanding of the fundamental biology of the cell membrane, a non-destructive method for evaluating intrinsic membrane physicochemical properties is required. As an example, packing or molecular order of membrane lipids can be sensed by fluorescent polarity-sensitive probes such as Laurdan or Di-4-ANEPPDHQ, whose emission spectrum shifts in response to changes in the molecular order of the membrane environment, usually quantified by a parameter denoted Generalized Polarization (GP) [6–11]. With the advent of commercial microscopes equipped with spectral detectors, shifts in the fluorescence emission spectra, and thus the GP parameter, can now be determined with much higher spatial accuracy using spectral imaging [10]. Owing to the internalization of many polarity-sensitive fluorescent probes in living cells, however, membrane segmentation must be performed to accurately measure membrane lipid packing and to remove cytosolic contributions [7, 12]. Membrane segmentation is often performed using a secondary fluorophore which increases experimental cost and complexity.

To this end, we have developed the Spectral Imaging Toolbox, a toolbox for spectral analysis with reliable membrane segmentation without the need for a secondary imaging probe. In the Spectral Imaging Toolbox, we have included batch and hyperstack processing as well as 3D reconstruction of confocal z-stacks to facilitate processing of large datasets and experiments with multiple exposures. We demonstrate the utility of this tool with images of giant plasma membrane vesicles (GPMVs, cell-derived vesicles) labelled with either polarity-sensitive Laurdan or Di-4-ANEPPDHQ, images of live cancer cells and microbubbles labelled with carboxyl-modified Laurdan (C-Laurdan), and giant unilamellar vesicles (GUVs) labelled with Di-4-AN(F)EPPTEA (FE). In addition to the more commonly employed Laurdan and Di-4-ANEPPDHQ dyes, we chose FE and C-Laurdan for their superior photostability and emission spectrum range [8, 12].

## Implementation

The Spectral Imaging Toolbox was designed for spectral analysis of high magnification images of single or sub-confluent cells, vesicles and microbubbles in MATLAB [13].

### Inputs and outputs

In spectral imaging, a stack of images of a sample region is recorded with each image in the stack monitoring a different wavelength range, such that the information from the whole stack discloses the spectrum of emitted fluorescence for each image pixel [10]. The Spectral Imaging Toolbox is designed for batch processing and 3-4D stacks. Using the Spectral Imaging Toolbox, we were able to process and analyze a dataset containing over 1500 cells in a few hours [3]. To our knowledge, this is the largest study using the GP parameter of cell membranes as a metric for membrane lipid order, highlighting the utility of our toolbox. For an input directory of spectral image stacks, the Spectral Imaging Toolbox outputs pseudo-colored GP maps, fitted GP histograms, and plotted spectra at the whole image, whole object, and segmented membrane levels for each image in the folder, as well as a spreadsheet summarizing the results. Input images and metadata are automatically converted to the OME-TIFF data standard using the Bio-Formats Library (144 image formats currently supported) [14]. Options for automatic 3D reconstruction of confocal spectral z-stacks [15] and plotted size distributions of spherical vesicles are also available.

### Graphical user interface (GUI)

A graphical user interface (GUI) guides the user through the analysis such that no programming skills are required. The GUI has a three panel design whereby the left panel displays instructions and menu items, the center panel allows for navigation through the images and user interaction (i.e., cropping and region of interest selection), and the right panel displays a gallery of images providing an overview of the results. The processing allows for user interaction at three steps. First, the user selects settings for which to run the Spectral Imaging Toolbox, such as whether to include membrane segmentation or a GP correction factor. Then following automatic object detection, the user has the option to segment each detected object further using one or more of several segmentation routines. Finally, the user can review the results and remove unwanted objects from the analysis as necessary.

### Segmentation

Spectral image stacks are thresholded using an intensity threshold determined automatically by Otsu’s method [16]. Objects of interest are then segmented and cropped using connected-component labelling of the binary thresholding mask [17]. The resultant cropped images are displayed for the user to discard off-target cropped images as necessary.

**Fig. 1:**
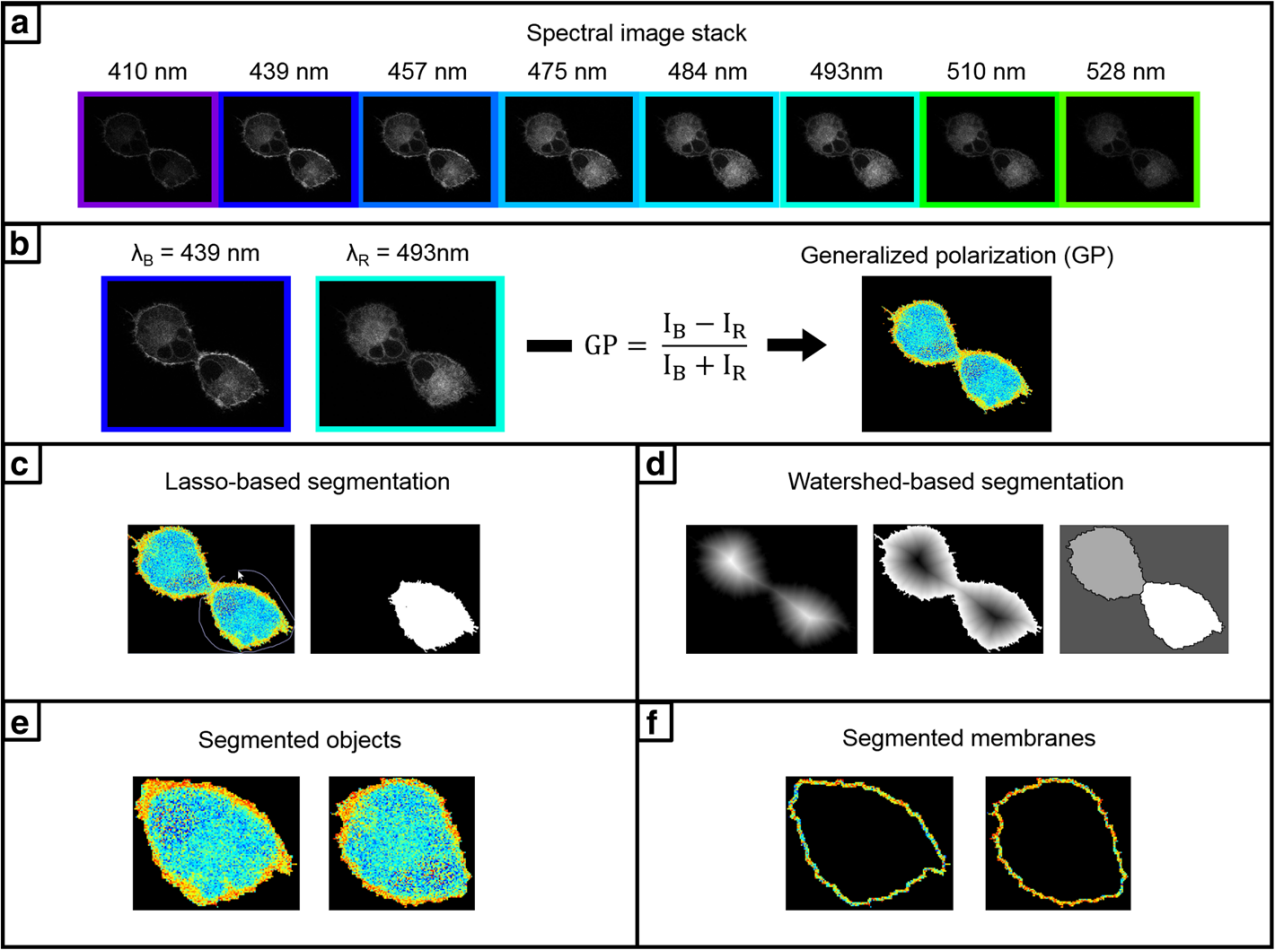
The Spectral Imaging Toolbox. **a** An auto-thresholded spectral image stack containing images of C-Laurdan fluorescence emission from labelled A-549 cells collected at wavelengths ranging from 410 to 528 nm. **b** Generalized polarization (GP) is then calculated at each pixel using the intensities (I_B_ and I_R_) from the images collected at λ_B_ and λ_R_ (*left*) using the equation (*center*). Pseudocolored GP maps can then be generated (*right*, color bar same as Fig. 2). Segmentation can then performed on the GP maps using lasso-based segmentation (**c**), where the user draws a region-of-interest (ROI) (*left*) used to generate a segmentation mask (*right*). Segmentation can alternately be performed using a watershed-based approach (**d**). From *left* to *right* in (**d**), the distance transform, the negated distance transform, and the labelled components following the watershed transform. Either segmentation routine will result in the segmented objects (**e**), from which a given number of border pixels are taken as the segmented membranes (**f**)

If a cropped image contains connected objects, such as fused GUVs or touching cell membranes, the user can readily separate them using a watershed-based segmentation approach. Prior to taking the watershed transform which identifies objects as catchment basins separated by watershed lines [18], a series of operations are conducted to improve performance. Namely, the distance transform of the complement of the binarized image is computed. The watershed transform of the negated distance transform is then taken. This process is demonstrated in Fig. 1d. By our method, the threshold level for suppressing shallow minima in this image is chosen such that the watershed transform labels *n* objects for segmentation, where *n* is input through the GUI. In other words, if the user specifies that a cropped image contains *n* cells, *n* cells are segmented. Owing to the sensitivity of this method to non-convex shaped cells and intracellular intensity variations, the user also has the option to segment manually using lasso-segmentation. Furthermore, lasso-segmentation can be used to conduct spectral analysis on any user-defined region of interest, including intracellular vesicles.

Since the cropped images contain only a single object, the membrane segmentation is simple and reliable. The objects in the binarized cropped images are filled and the membranes detected using Sobel edge detection [19–21]. The membranes are then segmented using the edge-detected pixels following dilation with a horizontal line element [22]. The Spectral Imaging Toolbox also has a spherical object mode designed for microbubbles and spherical vesicles, where objects are segmented by finding circles using the circular Hough transform [23, 24].

### Generalized polarization (GP)

As highlighted before, the GP parameter is introduced for quantification of the spectral shift in emission of a polarity-sensitive probe due to differences in lipid membrane order. GP is commonly calculated using fluorescence intensities collected at two emission wavelengths, λ_B_ and λ_R_, occurring at the emission maxima of the probe in a liquid-ordered and liquid-disordered reference solution respectively [9, 10]. GP, which varies from -1 to 1, is calculated for each pixel in the spectral image from the following equation,

1 $$ G P=\frac{I_B-{I}_R}{I_B+{I}_R}, $$

where *I*_*B*_ and *I*_*R*_ correspond to the fluorescence intensity at λ_B_ and λ_R_ emission wavelengths, respectively. Consequently, low GP values indicate more disordered environments.

To clarify, only the intensities of the images at λ_B_ and λ_R_ are required for GP calculation, even with spectral image stacks consisting of images collected at many wavelengths (e.g. Fig. 1a). Thus, the spectral image stack is reduced to two images at λ_B_ and λ_R_, and these two images are reduced to the single-valued GP parameter at each pixel (Fig. 1b).

The calculated GP values are then visualized using a pseudo-colored map with a look-up table scaled from -1 to 1 [11]. Finally, the distribution of GP values is fitted to either a one or two-peak Gaussian chosen by the lower root-mean squared error. The resultant GP histogram can be used to calculate changes in mean lipid order or, for a well-defined two-peak Gaussian, to indicate the presence of two phases [6]. To facilitate additional spectral analysis, spectra are generated from the mean intensities of images at each wavelength of the stack.

### Generalized polarization (GP) correction factor

As GP is an intensity-based measurement, it is strongly influenced by microscope settings including detector gain and filter settings. When GP is calculated using intensities I_B_ and I_R_ from two channels detecting at wavelengths λ_B_ and λ_R_, respectively, the relative intensities of the two channels must be calibrated to obtain absolute GP values. By acquiring an image of a reference solution with corresponding GP also measured with a fluorimeter (GP_ref_), a correction factor, G, can be introduced,

2 $$ G=\frac{I_{B,\  ref}\times \left(1- G{P}_{ref}\right)}{I_{R,\  ref}\times \left(1+ G{P}_{ref}\right)}, $$

where I_B,ref_ and I_R,ref_ correspond to the fluorescence intensity of the microscope image at λ_B_ and λ_R_ emission wavelengths, respectively [25]. GP is then calculated as follows,

3 $$ G P=\frac{I_B- G\times {I}_R}{I_B+ G\times {I}_R}. $$

In the Spectral Imaging Toolbox, GP_ref_ and a reference image can be specified in order to determine G for subsequent GP calculations.

## Results

Here we present several examples of spectral imaging data processed with the Spectral Imaging Toolbox.

### Spectral imaging by confocal microscopy

Spectral imaging was performed on a Zeiss LSM 780 confocal microscope equipped with a 32-channel gallium arsenide phosphide (GaAsP) detector array, as reported previously [10]. Laurdan, C-Laurdan, FE, and Di-4-ANEPPDHQ were excited at 405, 405, 488 and 488 nm respectively and the lambda detection ranges set between 410 nm and 695 nm, 415 nm and 691 nm, 500 nm and 650 nm, and 490 nm and 695 nm respectively. The resulting spectral image stacks were processed and analyzed using the Spectral Imaging Toolbox.

### Sample preparation

A-549 cells, immortalized human alveolar adenocarcinomic epithelial cells, were grown in standard culture conditions with Dulbecco’s modified eagle medium (DMEM) containing 10% fetal bovine serum (FBS) and 1% penicillin/streptomycin. Giant unilamellar vesicles (GUVs) made of dioleoyl phosphatidylcholine (DOPC), brain sphingomyelin (brain SM), and cholesterol from Avanti Polar Lipids were produced in a 2:2:1 molar ratio by electroformation by a modification of the protocol proposed by Angelova et al. [10, 26]. Phospholipid shelled microbubbles with a 9:1 molar ratio of 1,2-Distearoyl-sn-glycero-3-phosphocholine (DSPC, Avanti Polar Lipids, USA) and polyoxyethylene (40) stearate (PEG40S, Sigma Aldrich, UK) were produced using a batch sonication protocol previously reported [27]. Samples were labelled with either C-Laurdan (400 nM for A-549 cells and 100 nM for GUVs and microbubbles) or Di-4-AN(F)EPPTEA (FE) (100 nM for GUVs) in phosphate-buffered saline (PBS). Giant plasma membrane vesicles (GPMVs) were isolated from rat basophilic leukemia cells labelled with 100 nM Laurdan or 100 nM Di-4-ANEPPDHQ as described by Sezgin et al. [28]. Briefly, cells were exposed for 1 h at 37 °C to GPMV buffer (10 mM HEPES, 150 mM NaCl, 2 mM CaCl_2_, pH 7.4) containing 25 mM paraformaldehyde and 2 mM dithiothreitol for inducing vesiculation. After vesiculation, the GPMV-rich supernatant was collected by pipetting and resuspended in GPMV buffer for imaging. For all samples, spectral imaging was performed with samples on 170 μm thick glass coverslips.

### Segmentation of cells, GUVs, and microbubbles

**Fig. 2:**
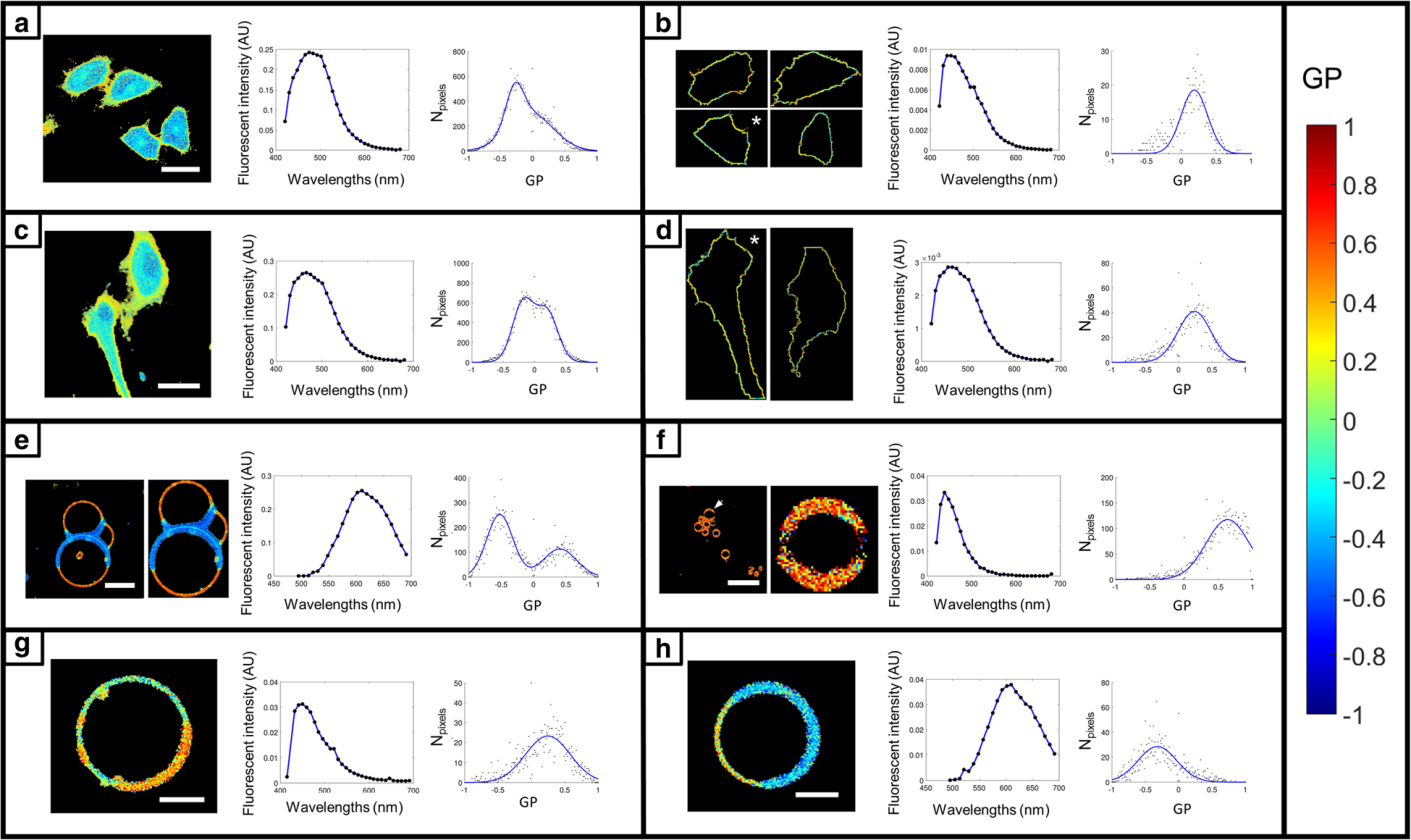
Segmentation and spectral analysis with the Spectral Imaging Toolbox. Each panel contains from *left* to *right*: a pseudo-colored GP map, the spectra calculated from all pixels of the spectral image stack with significant signal values, and a histogram of GP values fitted with either a single or double peak Gaussian. **a** A-549 cells stained with C-Laurdan, labeling both the plasma membrane and the cytosol. Scale bar 27 μm. **b** The same cells from (**a**) but now surface-segmented for plasma membrane only using the watershed method. **c** A-549 cells stained with C-Laurdan, labeling both the plasma membrane and the cytosol. Scale bar 33 μm. **d** The same cells from (**c**) but now surface-segmented for plasma membrane only using lasso-segmentation. In (**b**) and (**d**) the GP histograms and spectra are for the images indicated with an asterisk. **e** GUVs composed of DOPC, brain SM, and cholesterol (2:2:1 molar ratio) labelled with FE (Di-4-AN(F)EPPTEA). Unsegmented (*far left*), cropped and isolated GUVs in the adjacent image. Scale bar 17 μm. **f** GP image of C-Laurdan-labelled microbubbles (*far left*) was auto-segmented using the spherical object mode of the Spectral Imaging Toolbox. Scale bar 13 μm. One of the microbubbles, indicated by the arrow in the far *left* image, is shown post-segmentation in the adjacent image. Due to few pixels in the segmented microbubble, the GP distribution is shown for the unsegmented image. **g** GPMV labelled with Laurdan. Scale bar 5 μm. **h** GPMV labelled with Di-4-ANEPPDHQ. Scale bar 5 μm. Color bar legend gives GP values and is valid for all images

Image segmentation and spectral analysis using the Spectral Imaging Toolbox are demonstrated in Fig. 2. Pseudo-colored Generalized Polarization (GP) maps, fluorescence spectra generated from the mean intensities of images at each wavelength of the spectral image stack, and histograms of GP values fitted with either a single or double peak Gaussian are provided for each example. Spectral analysis is demonstrated with images of cells stained with C-Laurdan (Fig. 2a and c) and segmented by either the watershed method (Fig. 2b) or manually by lasso-segmentation (see Fig. 2d). The value of membrane segmentation in spectral analysis is highlighted by comparing the spectra and GP distributions of the segmented cells in Fig. 2b and d with the pre-segmentation results in Fig. 2a and c. The segmented spectra are blue-shifted and the GP increased reflecting the higher lipid order of cell membranes compared to the intracellular milieu. This is also indicated by the double-peak Gaussian GP distributions in the pre-segmentation results in Fig. 2a and c. Spectral analysis is also demonstrated with images of GUVs composed of a mixture of DOPC, brain SM, and cholesterol (2:2:1 molar ratio) labelled with FE (Fig. 2e). The presence of DOPC, brain SM, and cholesterol clearly give rise to phase separation as indicated by the distinct peaks in the GP histogram and in the pseudo-colored GP map. The Spectral Imaging Toolbox was used to auto-crop the fused GUVs and remove background objects for spectral analysis. In this case, membrane segmentation was not necessary because the interior of the GUVs was not fluorescent like in the examples with cells. The lower lipid order region on the GP map (blue pixels), however, is thicker due to this region having higher fluorescence intensity. Membrane segmentation could be used to take an equal-thickness sampling of pixels around the GUV, for consistency of analysis across a population of multiple GUVs. Vesicles derived from cell membranes (GPMVs) and labelled with Laurdan (Fig. 2g) or Di-4-ANEPPDHQ (Fig. 2h) also exhibit phase separation as indicated by their respective GP maps. The GP histograms from the GUVs and GPMVs illustrate a key difference between these two constructs. The phases present in GPMVs are much closer in lipid order than those present in GUVs. Spectral analysis with the spherical object segmentation mode of the Spectral Imaging Toolbox is demonstrated in Fig. 2f with an image of C-Laurdan-labelled microbubbles. The automated segmentation of a microbubble from a cluster of microbubbles is demonstrated.

### 3D reconstruction of pseudo-colored GP maps

**Fig. 3:**
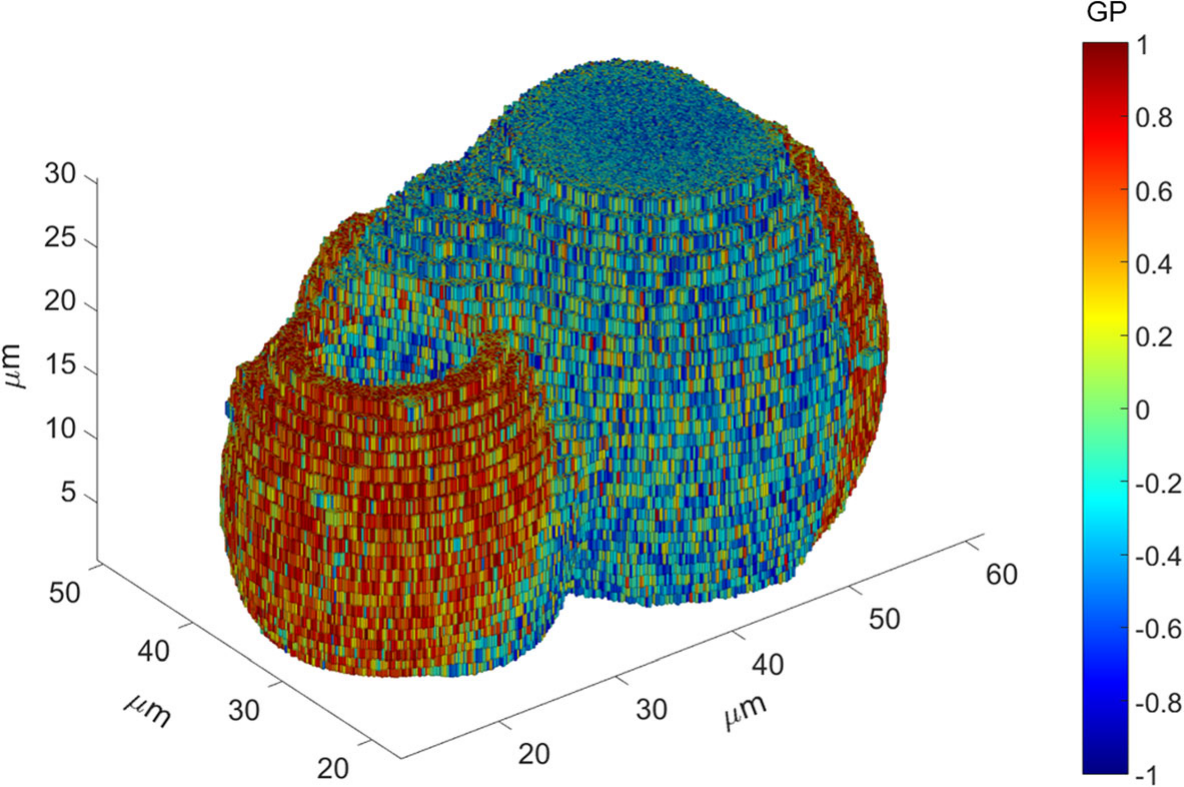
3D reconstructed GP image calculated from a spectral image stack of FE-labelled GUVs using the Spectral Imaging Toolbox. Axes give spatial dimensions along all three dimensions and color bar legend indicates GP values

A 3D reconstruction of pseudo-colored GP values calculated from a spectral z-stack of FE-labelled GUVs is demonstrated in Fig. 3. A single slice of the stack can be seen in Fig. 2e. The two phase-separated GUVs in the foreground are connected at their lower end through more lipid-ordered domains (GP < 0).

### Microbubble size distribution

**Fig. 4:**
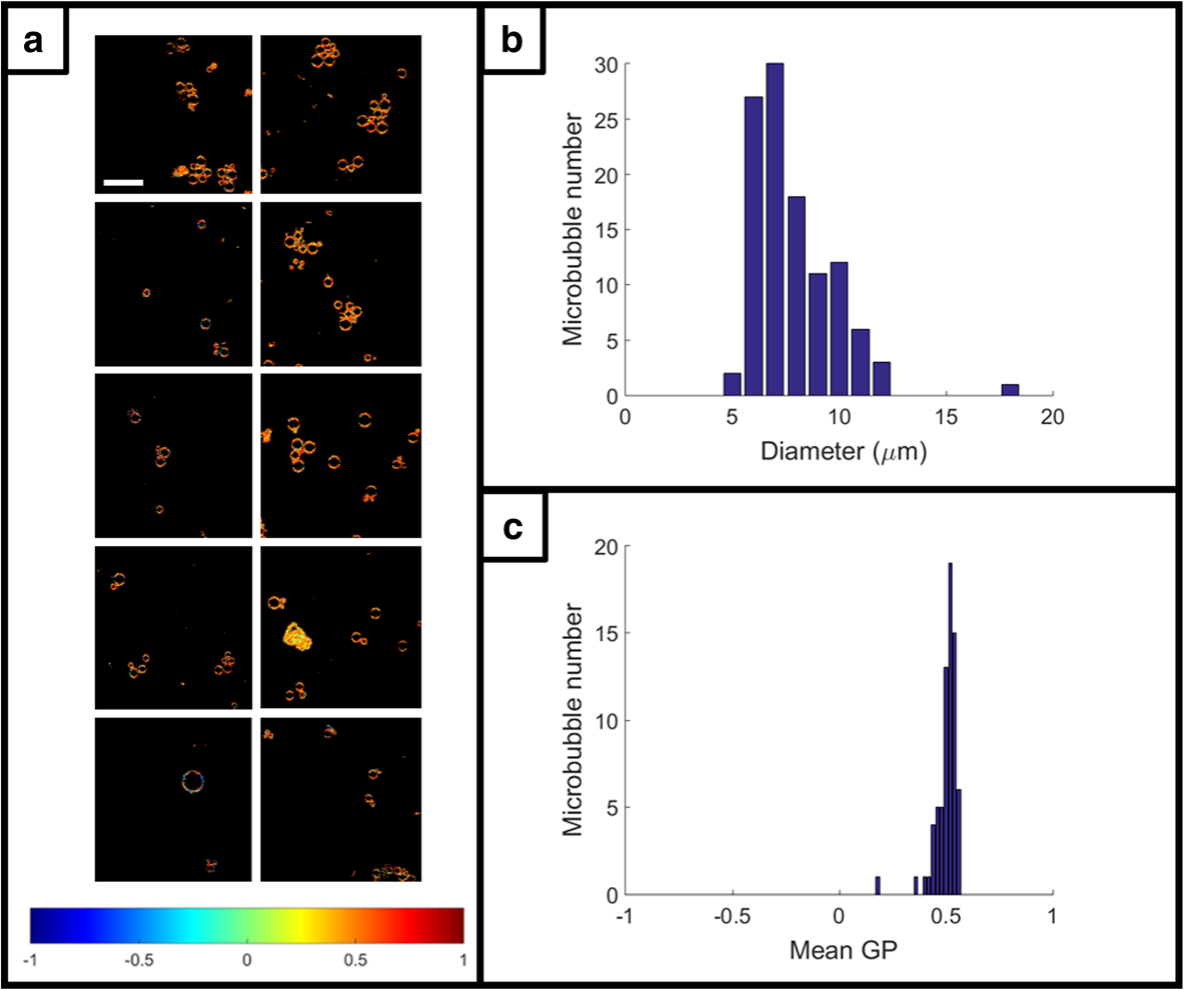
Spectral analysis and size distribution of microbubbles. **a** Pseudo-colored GP images from 10 spectral image stacks of microbubbles labelled with C-Laurdan. Microbubbles were auto-segmented and analyzed using the spherical object mode of the Spectral Imaging Toolbox. Scale bar 30 μm. Color bar legend gives GP values. **b** Size distribution (diameter) of the segmented microbubbles (*n* = 71). **c** Distribution of mean GP values for the segmented microbubbles (*n* = 71)

Ten spectral image stacks of DSPC-PEG 9:1 molar ratio microbubbles labelled with C-Laurdan were analysed with the spherical object segmentation mode of the Spectral Imaging Toolbox. The resultant pseudo-colored GP maps, size distribution of segmented microbubbles (*n* = 71), and distribution of mean GP values for the segmented microbubbles (*n* = 71) are displayed in Fig. 4.

## Discussion

### Novel aspects

The Spectral Imaging Toolbox is the first free and open-source software to accurately measure cell membrane lipid packing without cytosolic contributions using a single dye. Furthermore, by implementing batch and hyperstack processing as well as 3D reconstruction of confocal z-stacks, it addresses a growing need to process large spectral imaging datasets and data from experiments with multiple exposures. It is also the only spectral imaging software to our knowledge to leverage different processing routines for vesicles, for adherent cells, and for regions of interest (i.e., sub-cellular) respectively. Finally, while the algorithms used are not individually novel, their implementation for spectral imaging is not available elsewhere to our knowledge.

### Comparison with existing software

Without using membrane segmentation, it is common to decompose the GP histogram into two Gaussian components whereby the lower GP component corresponds primarily to the intracellular regions and the higher GP component to the cell membrane [29]. While this technique is valuable for localizing high and low lipid order regions, it is not appropriate for determining plasma membrane lipid order. Low lipid order domains in the membrane and high lipid order vesicles inside the cell, for instance, could not be attributed to their respective sub-cellular components without some form of segmentation. Thus, more advanced software is required for accurately determining membrane lipid order.

Existing tools of note for processing spectral imaging data with the GP parameter include the ImageJ plugins of Sezgin et al. and Owen et al., and SimFCS developed by Professor Enrico Gratton [10, 30, 31]. These tools all provide adequate means of calculating GP, generating GP visualizations, and histograms for a single spectral image.

The plugin of Owen et al. provides batch processing and enables membrane segmentation with the requirement of a secondary image acquisition and fluorescent membrane label. The Spectral Imaging Toolbox does not require an additional membrane label or image acquisition step to achieve membrane segmentation.

Sezgin et al. allow for fitting the spectra of each pixel with either a Gaussian or gamma-variate function to interpolate the intensities, I_B_ and I_R_, for reducing noise in the GP calculation. We found that the gamma-variate fit is most appropriate for spectral imaging data but was too computationally expensive for batch and hyperstack processing. The Spectral Imaging Toolbox instead allows for optionally smoothing the intensity images using a median filter prior to GP calculation, much like SimFCS.

The power of SimFCS is its ability to process many types of advanced imaging data with one software suite. SimFCS does not, however, support batch processing, ROI segmentation, membrane segmentation, or z-stack GP analysis and visualization - core features of the Spectral Imaging Toolbox.

Regarding availability, ImageJ is free [32], as is SimFCS 2 from Globals Software (although the laboratory license for the updated version, SimFCS 4, is $2000). Most research institutions have MATLAB licenses and without a site license, students can purchase MATLAB with the necessary add-ons for only $60.

Another benefit of our software is the ease of customization. SimFCS is not designed for user modification of the source code, and ImageJ provides only a limited macro language and plugin facility. Conversely, the Spectral Imaging Toolbox can be readily extended using MATLAB vector operations well-suited to rapid and complex image processing and analysis. The open-source code will be maintained on the MATLAB Central File Exchange at the URL provided where updates and feature requests can be publicly discussed.

## Conclusion

The Spectral Imaging Toolbox provides an easy-to-use means of analyzing large spectral imaging datasets. It requires no programming experience, outputs publication-quality figures, enables reliable membrane segmentation without the requirement of a counter stain, and incorporates batch and hyperstack processing. It is our intention to continue to develop this free and open-source toolbox with input from the community to further facilitate ambitious research with spectral imaging.

## Availability and requirements

Project name: Spectral Imaging Toolbox

Project web page: https://ora.ox.ac.uk/objects/uuid:4375842f-3598-418d-8aa3-9b31f5023401

Operating system: Tested on Windows 7

Programming language: MATLAB 2015+

Other requirements: Image Processing Toolbox https://uk.mathworks.com/matlabcentral/fileexchange/62617-spectral-imaging-toolbox

License: GPL

Any restrictions on use by non-academics: none

## References

[1] Lingwood D, Simons K (2010). Lipid rafts as a membrane-organizing principle. Science.

[2] Simons K, Gerl MJ (2010). Revitalizing membrane rafts: new tools and insights. Nat Rev Mol Cell Biol.

[3] Carugo D, et al. Modulation of the molecular arrangement in artificial and biological membranes by phospholipid-shelled microbubbles. Biomaterials. 2016;113:105. 10.1016/j.biomaterials.2016.10.034 27814482

[4] Hosny NA (2013). Mapping microbubble viscosity using fluorescence lifetime imaging of molecular rotors. Proc Natl Acad Sci U S A.

[5] Lentacker I (2014). Understanding ultrasound induced sonoporation: Definitions and underlying mechanisms. Adv Drug Deliv Rev.

[6] De La Serna Bernardino J (2013). Compositional and structural characterization of monolayers and bilayers composed of native pulmonary surfactant from wild type mice. Biochim Biophys Acta.

[7] Dodes Traian MM (2012). Imaging lipid lateral organization in membranes with C-laurdan in a confocal microscope. J Lipid Res.

[8] Kwiatek JM (2013). Characterization of a new series of fluorescent probes for imaging membrane order. PLoS One.

[9] Parasassi T (1997). Two-photon fluorescence microscopy of laurdan generalized polarization domains in model and natural membranes. Biophys J.

[10] Sezgin E (2015). Spectral imaging to measure heterogeneity in membrane lipid packing. ChemPhysChem.

[11] Yu W (1996). Fluorescence generalized polarization of cell membranes: a two-photon scanning microscopy approach. Biophys J.

[12] Sezgin E (2014). Measuring lipid packing of model and cellular membranes with environment sensitive probes. Langmuir.

[13] MATLAB (2015). version 8.5.0 (R2015a) The Mathworks Inc., Natick, Massachusettes.

[14] Linkert M (2010). Metadata matters: access to image data in the real world. J Cell Biol.

[15] Aitkenhead A. Plot a 3D array using patch. MATLAB Central File Exchange. 2010. https://www.mathworks.com/matlabcentral/fileexchange/28497-plot-a-3d-array-using-patch. Retrieved March 25, 2016.

[16] Otsu N (1979). A threshold selection method from gray-level histograms. IEEE Trans Syst Man Cybern.

[17] Haralock RM, Shapiro LG (1991). Computer and robot vision Addison-Wesley Longman Publishing Co., Inc.

[18] Meyer F (1994). Topographic distance and watershed lines. Signal Process.

[19] Lim JS (1990). Two-dimensional signal and image processing.

[20] Parker JR (2010). Algorithms for image processing and computer vision John Wiley & Sons.

[21] Soille P (2013). Morphological image analysis: principles and applications Springer Science & Business Media.

[22] van den Boomgaard R, van Balen R (1992). Methods for fast morphological image transforms using bitmapped binary images. CVGIP Graph Model Image Process.

[23] Atherton TJ, Kerbyson DJ (1999). Size invariant circle detection. Image Vis Comput.

[24] Yuen H (1990). Comparative study of Hough Transform methods for circle finding. Image Vis Comput.

[25] Brewer J (2010). Multiphoton excitation fluorescence microscopy in planar membrane systems. Biochim Biophys Acta.

[26] Angelova MI, Dimitrov DS (1986). Liposome electro formation. Faraday Discuss Chem Soc.

[27] Carugo D (2015). Biologically and acoustically compatible chamber for studying ultrasound-mediated delivery of therapeutic compounds. Ultrasound Med Biol.

[28] Sezgin E (2012). Elucidating membrane structure and protein behavior using giant plasma membrane vesicles. Nat Protoc.

[29] Golfetto O (2013). Laurdan fluorescence lifetime discriminates cholesterol content from changes in fluidity in living cell membranes. Biophys J.

[30] Owen DM (2012). Quantitative imaging of membrane lipid order in cells and organisms. Nat Protoc.

[31] Sanchez S a (2012). Laurdan generalized polarization fluctuations measures membrane packing micro-heterogeneity in vivo. Proc Natl Acad Sci.

[32] Schindelin J (2014). The ImageJ ecosystem: an open platform for biomedical image analysis. Mol Reprod Dev.

